# Integration of Developmental and Environmental Signals via a Polyadenylation Factor in Arabidopsis

**DOI:** 10.1371/journal.pone.0115779

**Published:** 2014-12-29

**Authors:** Man Liu, Ruqiang Xu, Carrie Merrill, Liwei Hong, Carol Von Lanken, Arthur G. Hunt, Qingshun Q. Li

**Affiliations:** 1 Department of Biology, Miami University, Oxford, OH 45045, United States of America; 2 Department of Plant and Soil Sciences, University of Kentucky, Lexington, KY 40506, United States of America; 3 Key Laboratory of the Ministry of Education on Costal Wetland Ecosystems, College of the Environment and Ecology, Xiamen University, Xiamen, Fujian 361102, China; 4 Rice Research Institute, Fujian Academy of Agricultural Sciences, Fuzhou, Fujian, 350003, China; Shanghai Center for Plant Stress Biology, Chinese Academy of Sciences, China

## Abstract

The ability to integrate environmental and developmental signals with physiological responses is critical for plant survival. How this integration is done, particularly through posttranscriptional control of gene expression, is poorly understood. Previously, it was found that the 30 kD subunit of Arabidopsis cleavage and polyadenylation specificity factor (AtCPSF30) is a calmodulin-regulated RNA-binding protein. Here we demonstrated that mutant plants (*oxt6*) deficient in AtCPSF30 possess a novel range of phenotypes – reduced fertility, reduced lateral root formation, and altered sensitivities to oxidative stress and a number of plant hormones (auxin, cytokinin, gibberellic acid, and ACC). While the wild-type AtCPSF30 (C30G) was able to restore normal growth and responses, a mutant AtCPSF30 protein incapable of interacting with calmodulin (C30GM) could only restore wild-type fertility and responses to oxidative stress and ACC. Thus, the interaction with calmodulin is important for part of AtCPSF30 functions in the plant. Global poly(A) site analysis showed that the C30G and C30GM proteins can restore wild-type poly(A) site choice to the *oxt6* mutant. Genes associated with hormone metabolism and auxin responses are also affected by the *oxt6* mutation. Moreover, 19 genes that are linked with calmodulin-dependent CPSF30 functions, were identified through genome-wide expression analysis. These data, in conjunction with previous results from the analysis of the *oxt6* mutant, indicate that the polyadenylation factor AtCPSF30 is a regulatory hub where different signaling cues are transduced, presumably via differential mRNA 3′ end formation or alternative polyadenylation, into specified phenotypic outcomes. Our results suggest a novel function of a polyadenylation factor in environmental and developmental signal integration.

## Introduction

The 3′-end processing of eukaryotic mRNA, that entails cleavage and polyadenylation of a precursor RNA, is a critical step of gene expression [1], [2]. The poly(A) tail promotes the translation and stability of the mRNA as well as the transport of mRNA from the nucleus to cytoplasm. In addition, the process of 3′-end formation is tightly coupled with transcriptional initiation and termination [3]. 3′-end processing is directed by polyadenylation signal sequences at 3′-end of precursor mRNA, and involves collaborations of a large number of protein factors. In mammals, the polyadenylation complex may be resolved into four major factors or multisubunit subcomplexes: Cleavage and Polyadenylation Specificity Factor (CPSF), Cleavage stimulation Factor (CstF), and Cleavage Factors I and II (CFI and CFII). In addition, poly(A) polymerase (PAP), symplekin, and the nuclear poly(A) binding protein (PABPN1) also play vital roles to 3′ end processing of mRNA [2], [4], [5], [6]. All those factors are required for cleavage reaction, but only CPSF, PAP, and PABP are necessary for adding poly(A) tails. CPSF recognizes the polyadenylation signal (typically AAUAAA) located in 10∼30 bp upstream of cleavage site [7]; recognition of G/U rich domain in downstream of cleavage site is mediated by CstF [8].

Several homologues of polyadenylation factors have been identified in Arabidopsis based on sequence similarities [1], [9]. Interestingly, mutations that affect the expression of different polyadenylation factor subunits have striking phenotypic consequences. The Arabidopsis orthologs of CPSF subunits CPSF100 and CPSF73 (AtCPSF100 and AtCPSF73-II) are essential proteins, and genetic manipulation of the expression of genes encoding these proteins reveals their roles in posttranscriptional gene silencing [10] and female gametophyte transmission efficiency [11], respectively. Other genetic studies have implicated AtPCFS4 and FY, two additional homologues of mammalian or yeast polyadenylation factor subunits, in the regulation of flowering time [12], [13]. The Arabidopsis orthologs of CstF77 and CstF64 help to control the expression of the flowering factor FLC through epigenetic regulations [14]. Proteins related to CstF64 and symplekin also contribute to epigenetic regulation in Arabidopsis [10]. Recent studies also imply that epigenetic marks may be linked to alternative polyadenylation regulation through other potential axillary proteins during polyadenylation process [15].

**Figure 1:**
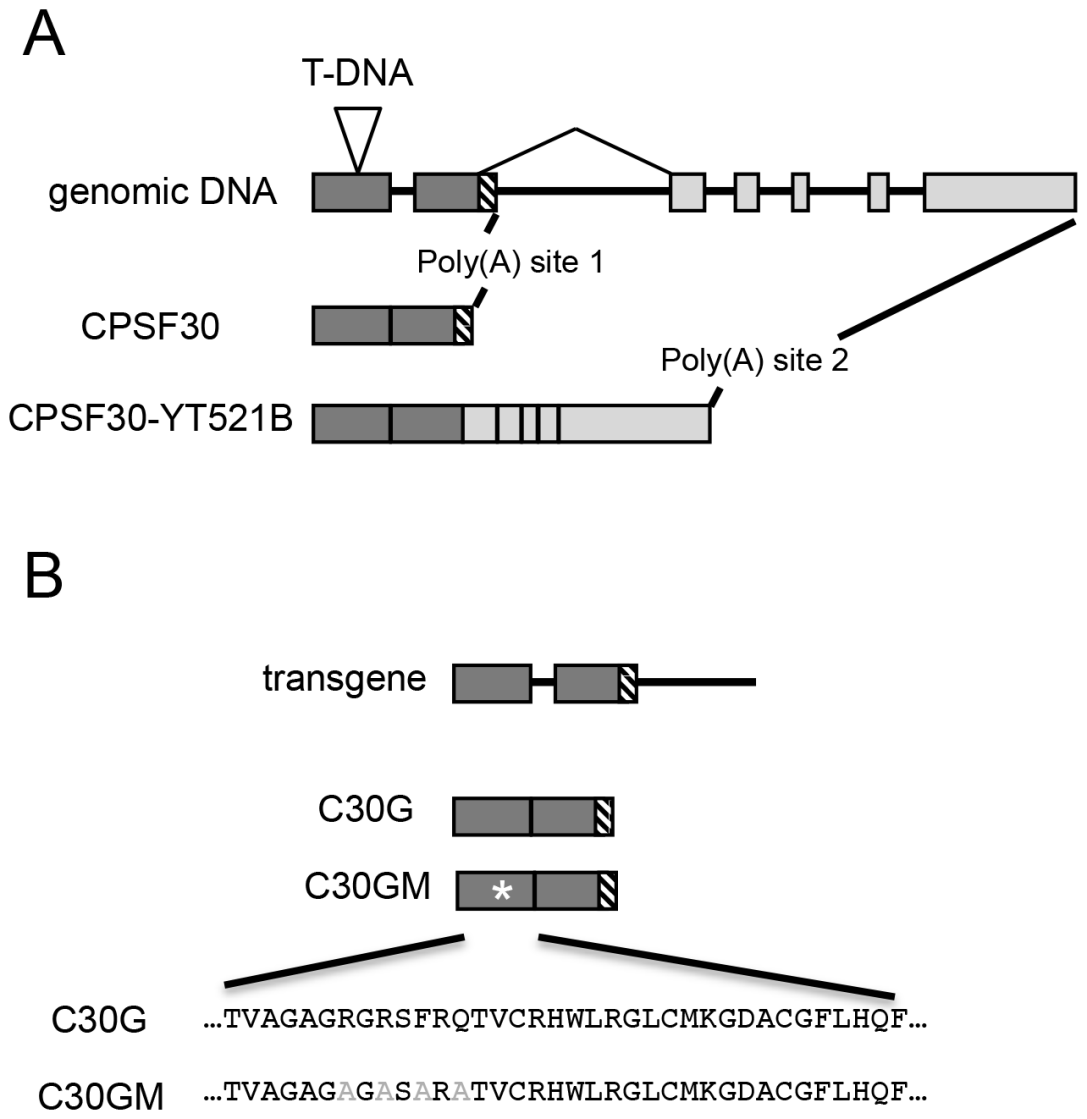
Structures of the transgenes assembled for this study, with an insert showing the wt (A) and mutant CAM-binding domain (B). A, the genomic DNA is shown at the top, and the two mRNAs beneath. Dark gray boxes indicate the exons present in *CPSF30*, the smaller of the two transcripts of the *OXT6* gene; light boxes are additional exons in *CPSF30-YT521B*, the larger of the two transcripts of *OXT6*. For brevity, and because the structures are identical for the mutation illustrated at the bottom, only the genomic DNA of the wild-type transgene is shown. The sequences at the bottom of B show the changes used to create the calmodulin-binding mutant; these have been described before [18].

One of the Arabidopsis polyadenylation factor subunits, AtCPSF30, has been implicated in the responses of plants to oxidative stress [16]. Recent research showed that AtCPSF30 is critical to plant immune response by regulating salicylic acid signaling pathway [17]. While the Arabidopsis protein is an ortholog of its mammalian (CPSF30) and yeast (YTH1p) counterparts, AtCPSF30 is different in interesting and important ways. AtCPSF30 is encoded by a locus that is subject to alternative splicing resulted in two proteins, AtCPSF30 and a larger protein of 375 amino acids suspected to be involved in splicing (Fig. 1A) [18]. CPSF30 is essential in other organisms, and directly interact with AAUAAA regulating mRNA 3′ processing through its zinc finger domains in animals [19]. However, an Arabidopsis mutant (*oxt6*) with a T-DNA insertion in the first exon of the gene (Fig. 1A) is viable. AtCPSF30 possesses a calmodulin-binding protein. Upon bound by calmodulin, AtCPSF30 loses its RNA-binding activity in a calcium-dependent manner [18].

The stress-tolerant phenotypes of the *oxt6* mutant, along with the calmodulin-binding capability of the protein, are characteristics that lend themselves to a model whereby intracellular calcium signals may affect AtCPSF30 functioning and thus phenotypic outputs. To explore this model, we have expressed wild-type and calmodulin-binding mutant forms of AtCPSF30 in *oxt6* and studied the physiological responses of various complemented plant lines. In so doing, we have found that *oxt6* has a number of hitherto-unidentified phenotypes. The characteristics of the various mutants and complemented lines indicate that the AtCPSF30-calmodulin interaction is required for some, but not all of the *in vivo* roles of the protein. Based on these and other studies, we propose that AtCPSF30 is a novel and versatile integrator of different cellular signaling cues, and that many growth processes involve regulated polyadenylation mediated by AtCPSF30.

## Materials and Methods

### Plant Materials and Growth Conditions

We obtained *Arabidopsis thaliana* Columbia (Col-0; CS6000) ecotype from the Arabidopsis Biological Resource Center, Columbus, Ohio, USA. The o*xt6* mutant has been described previously [16]. PCR amplification of the T-DNA flanking sequence was done by using primer P500 (5′-TGGTTCACG TAGTGGGCCATCG-3′) with the *AtCPSF30* gene-specific primers P258 (5′-CAGAACCCAATTAAAAACCTTAG-3′) to confirm T-DNA insertion position in *oxt6*. Plants were grown in Metro-Mix200 soil (Scotts-Sierra Horticultural Products Co.) at 22±2°C under a 16 h-light/8 h-dark cycle.

For the studies using defined media, seeds were sterilized with 20% bleach and 70% ethanol for 5 minutes respectively, and then washed five times in sterilized water. Sterilized seeds were germinated on half strength of Murashige and Skoog (MS) medium with 1% (W/V) sucrose and incubated in a vertical orientation in growth chambers at 22°C under constant light. Media were supplemented with 10 nM 6-benzylaminopurine (6-BA), 1 µM gibberellic acid (GA3), 10 nM indoleacetic acid (IAA), 25 µM (ACC; 1-aminocyclopropane-1-carboxylic acid), or 50 nM methyl viologen (MV) as indicated. Roots were analyzed after 10 days. For the lateral root data, the total numbers of lateral roots per primary root were tabulated. For the hormone and MV treatments, the total root lengths were measured and compiled. Between 10 and 30 individual plants were measured for each determination, and different biological samples assessed using the Student's t-test.

### Plasmid Construction and Plant Transformation

All the plasmids were constructed by using Gateway cloning technology (Invitrogen*)* [12], [20]. For the complementation experiments with the wild-type *AtCPSF30* gene, the 4.1 kb genomic sequence of the At1g30460 locus was amplified with primer pair P522 (5′-CACCATGGAACGCAATAGCTTTGATCC-3′) and P520 (5′-CGAGCAGTCAAGATGAGCATC-3′) using Arabidopsis Col-0 genomic DNA as a template. The amplified DNA fragment was first cloned into pTOPO-D vector (Invitrogen) to yield pTOPO-C30G. This plasmid was used to move the C30G sequences into the destination vector pMDC164 using the LR reaction [21] to yield pMDC123-C30G plasmid. For the complementation experiments with the calmodulin-binding AtCPSF30 mutant, the calmodulin-binding site in the AtCPSF30 coding region in pTOPO-C30G was modified using the QuickChange II XL-Site-Directed Mutagenesis Kit (Stratagene) with primer pair P512 (5′-CGGTGGCTGGAGCTGGGGCGGGTGCAAGTGCACGTGCAACTGTTTGTAGACACTGG-3′) and P513 (5′-CCAGTGTCTACAAACAGTTGCACGTGCACTTGCAC CCGCCCCAGCTCCAGCCACCG-3′). These primers introduce the same mutation that was described previously [18]; the mutant protein retains normal RNA-binding and endonuclease activities but is not inhibited by calmodulin. The resulting plasmid, pTOPO-C30GM, was used to move the C30GM sequences into the pMDC164 vector to yield pMDC123-C30GM. All clones were confirmed by DNA sequencing. Transgenic plants were identified by Basta screening, and homozygous plants were selected. 3–6 independent transgenic lines were selected, and the ones used/tested are listed in Figure S1 in S1 File.

### Gene Expression Analyses

For the quantitative real-time PCR analysis, total RNA was isolated from seedlings (16-day-old), using Invitrogen's Concert *Plant RNA Reagent* and treated with TURBO DNA-*free* DNase I (Invitrogen). 1.5 µg DNA-free total RNA was used for a reverse transcription reaction using oligo (dT)_20_ primer and Superscript III reverse transcriptase (Invitrogen). Quantitative real-time PCR was run on an iCycler (BioRad) using SYBR green PCR master mix. Primer pairs P1018 (5′-GTGAAAA CTGTTGGAGAGAAGCAA-3′) and P1019 (5′-TCAACTGGATACCCTTTCGCA-3′) for *TIP41-LIKE* (At4g34270), P1086 (5′- CCGCCTGAAAACTCTTCCT-3′) and P1087 (5′- TGAACCAATAACAACGTCTTGA-3′) for *AtCPSF30*, were used to detect the expression of *TIP41-LIKE*, and *AtCPSF30*. The expression of *TIP41-LIKE* was used as an internal reference to normalize the data [22].

### Phenotypic Analyses

For whole-mount observation, the roots were cleared and mounted as described [23]. Briefly, roots were fixed in 0.24N HCl with 20% methanol and incubated at 57°C for 15 minutes. After that, the solution was replaced to 7% NaOH, 7% hydroxylamine-HCl in 60% ethanol at room temperature for 15 minutes. Roots were then rehydrated through conventional ethanol series (40%, 20% and 10%) with 5 minutes each step, and cleared in 5% ethanol, 25% glycerol. Roots were soaked in 50% glycerol to observation by a Nomarski microscope (Zeiss).

To photograph flowers, flowers of appropriate stage were dissected under an Olympus AX-70 microscope and photographed. Image pro software was used to measure the length of pistil and stamens.

### Global poly(A) site analysis

Poly(A) site choice was profiled using RNA isolated from roots of the wt, *oxt6*, *oxt6*::C30G, and *oxt6*::C30GM lines. Total RNA were isolated from 10-day old roots via Qiagen RNeasy plant mini kit. 2 µg total RNA from each samples were fragmented in zinc buffer at 70°C for 5 min. RAN fragments with poly(A) tail were purified via poly(T) magnetic beads, and a 5′-end repair was performed using T4 polynucleotide kinase, then a DNA/RNA hybrid adaptor was ligated to the 5′end as an anchor. Next, the library was generated by reverse transcription with oligo(dT)-adapter followed by PCR amplification with Illumina adapters. The library was purified by gel isolation for a size selection of between 300 bp and 500 bp, and then purified by using QIAquick gel extraction kit. Detailed methods are described in Liu et al [24]. Subsequent computational analysis was performed using CLC Genomics Workbench, Excel, and the poly(A) profile comparison tool described [25]. Sequences in fastq format were imported into the CLC environment, demultiplexed into separate files corresponding to the four biological samples, and the sequences then trimmed to remove residual oligo-dT and Illumina adapter sequences. Each sequence set was mapped onto a custom reference database that consists of the set of Arabidopsis 3′-UTRs that had been extended at their 3′ ends by 500 nts; this extension is intended to improve the chances of recovering products of inefficient transcription termination. The database used for mapping consisted of the reverse complement of all extended 3′-UTRs; the rationale for this is given in [25]. Mapping results were exported as.sam files and used directly in the PATAPP program as described previously [25]. This program compares poly(A) site distributions in two samples on a gene-by-gene basis. The output from this analysis is, for each gene, a numeric value that represents the difference in poly(A) site profile in the two samples being compared. These values are displayed as a cumulative plot, and the relative poises of the respective curves (one for each pairwise comparison) defines the similarity (or differences) of poly(A) site choice genome-wide. The typical range, represented by the wt-*oxt6* comparison is from ref. 25 and used to assess the comparisons reported here.

All PAT-seq read files are available from the NCBI SRA database (accession # SRP050424).

### Digital gene expression (RNA-Seq) analysis

Gene expression was measured using the RNA-Seq tool in CLC Genomics Workbench, with the poly(A) tags serving as the data input for this. (Poly(A) tags such as generated in this study are suitable proxies for gene expression [26]). Tags that had been demultiplexed and trimmed were mapped to the Arabidopsis genome (TAIR10 version) using the default settings for RNA-Seq analysis. The total numbers of tags that mapped to individual genes were used to define expression levels. An initial normalization was done to convert expression values to tags per million (tpm). For subsequent MAPMAN analysis, genes that did not have expression levels greater than 5 tags per million in at least one sample (or plant line) were removed, and a value of 1 added to the remaining normalize. For the remaining genes, a value of 1 was added to each normalized expression value, the wt/*oxt6* expression ratios were determined and the log2 values for each ratio calculated. These latter values were used in the MAPMAN program [27], [28] to identify sets of functionally-related genes that, collectively, are affected by the *oxt6* mutation. Results were tabulated (S1 Table) and the uncorrected p-values for the Wilcoxon Rank Sum Test plotted.

Further RNA-Seq analysis was also done using CLC Genomics Workbench. For this, the same set of genes that had been normalized, filtered for expression, and adjusted (with the addition of 1 to each value) were further analyzed using Baggerly's test on proportions, using the default settings of the software package. The filters described in S2 Table were used to identify candidate genes whose expression is affected by the mutation of the calmodulin-binding domain of AtCPSF30; these genes are described in S2 Table.

## Results

### The Interaction between AtCPSF30 and Calmodulin Is Not Required for the Functioning of AtCPSF30 in Responses to Oxidative Stress

Previously, an Arabidopsis mutant bearing a T-DNA insertion within the first exon of the gene (*OXT6*) that encodes the Arabidopsis CPSF30 (AtCPSF30; see Fig. 1A) was identified in a screen for mutants with enhanced tolerance to oxidative stresses [16], [18]. In this mutant, no mRNAs capable of encoding AtCPSF30 could be detected, and no AtCPSF30-related polypeptides (either of the isoforms depicted in Fig. 1A) could be detected [16], [18]. Given that rapid calcium fluxes seem to contribute to the responses of plants to treatments that also involve the generation of reactive oxygen species, it seemed possible that the role of AtCPSF30 in responses to oxidative stress may involve its interaction with calmodulin. Accordingly, transgenes that encode the wild-type AtCPSF30 (C30G) or a mutant deficient in its interaction with calmodulin (C30GM) were introduced into the *oxt6* mutant; the nature of these transgenes and of the mutant that does not bind calmodulin is shown in Fig. 1B. Several independent homozygous lines were produced, and the transgene expressions were screened by quantitative RT-PCR (Figure S1 in S1 File); lines representing a range of expression levels were chosen for subsequent studies.

**Figure 2:**
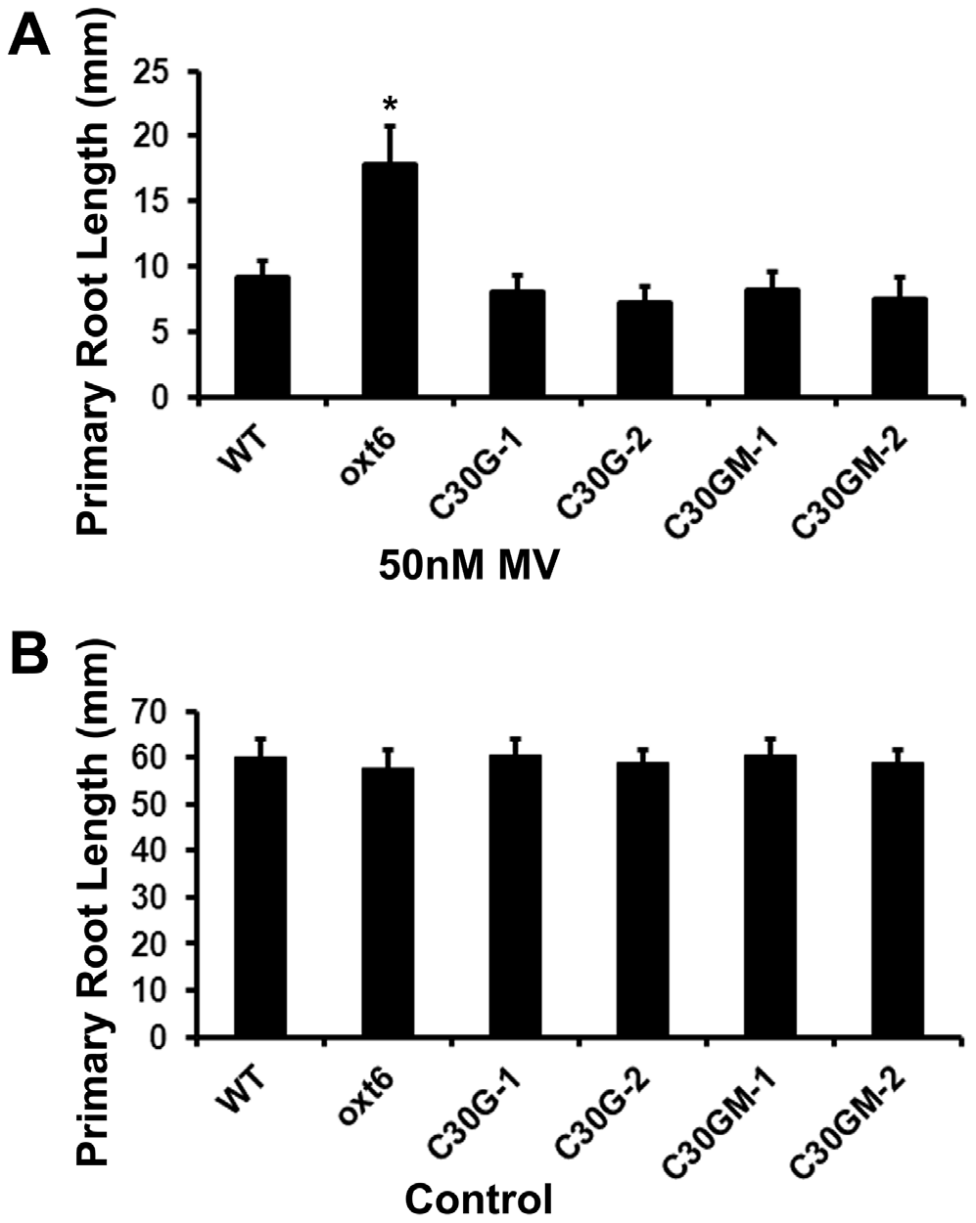
Root length assay of the response of the *oxt6* mutant to methyl viologen. (A) Methyl violgen treatment. Plants with the indicated genotypes were grown as described in the Methods on media containing 50 nM methyl viologen; root lengths were measured after 10 days of growth. (B) Without methyl violgen treatment. The same growth condition as in A but without added methyl viologen. Wt, the wild-type parent of the *oxt6* mutant; oxt6, the *oxt6* mutant; C30G-1, the C30G-1 line whose expression is assessed in Figure S2 in S1 File; C30G-2, line C30G2-2 in Figure S2 in File S1; C30GM-1, line C30GM-1 in Figure S2 in S1 File; C30GM-2, line C30GM-2 in Figure S2 in S1 File. Error bars denote standard deviations; * indicates a difference from the wild-type with a p-value <0.05 (Student's t-test, n≥12).

To elucidate the responses of these transgenic lines to oxidative stress, the lengths of roots of seedlings grown on defined media containing 50 nM methyl viologen (MV) were determined; this assay is the same as that used in the previous characterization of the mutant [16]. As was seen before [16], the *oxt6* mutant roots grew much better on MV than did the wild-type (Fig. 2A); on control plates lacking MV, the root growth of wt and *oxt6* mutant seedlings was indistinguishable (Fig. 2B). In contrast, plants that express the wild-type AtCPSF30 (C30G-1 and C30G-2; gene construct in Fig. 1B and expression level in Figure S1 in S1 File) showed wild-type sensitivity to MV (Fig. 2A). These results corroborate the earlier result that indicates that the enhanced growth of the mutant on MV is due to a deficit in AtCPSF30 expression [16]. Plants that express the calmodulin-binding mutant (C30GM-1 and C30GM-2; gene construct in Fig. 1B and expression level in Figure S1 in S1 File) also showed wild-type sensitivity to MV (Fig. 2A). The growth of the C30G and C30GM plants on control plates was similar to that seen with the wild-type (Fig. 2B). These results indicate that the C30G and C30GM transgenes are expressed at sufficient levels to permit complementation of the MV phenotype, and that both transgenes encode functional proteins. They also show that the interaction between AtCPSF30 and calmodulin is not required for wild-type sensitivity of plants to MV.

### The Interaction between AtCPSF30 and Calmodulin Is required for the Functioning of AtCPSF30 in Lateral Root Development

**Figure 3:**
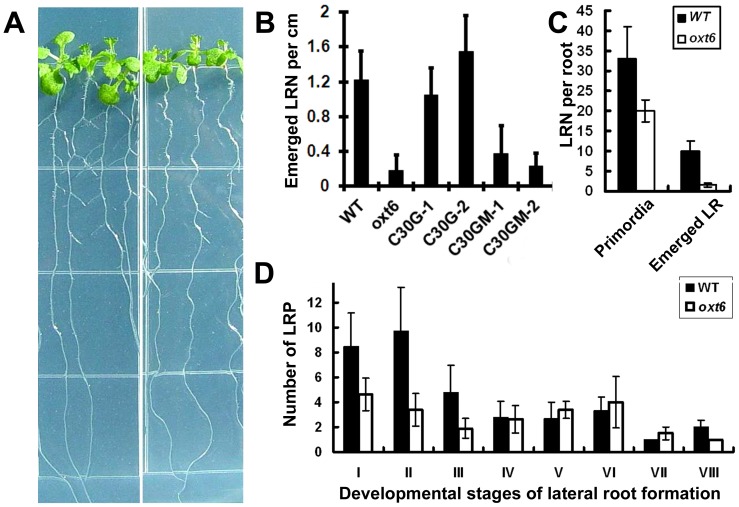
Lateral root development of the *oxt6* mutant and complemented plants. (A) Image of 10-days-old wild-type (wild-type, left) and *oxt6* (right) seedlings grown on a half MS medium plate. (B) Numbers of lateral roots per primary root per cm in wild-type, *oxt6, oxt6*::C30G, and *oxt6*::C30GM lines. Plants were germinated and grown on vertical plates for 10 days, when lateral roots were counted. (C) Primordia in the wild-type and *oxt6* mutant. Primordia were counted on whole-mount roots of wild-type and *oxt6* plants 10 days after germination and growth on vertical plates. (D) Numbers of primordia at the different stages of lateral root development (as defined in [23]). *n*>8 plants per column.

In the course of performing further assays of the sensitivity of the wild-type and mutant to MV, it was noted that the root systems of the mutant seemed to possess fewer lateral roots (Fig. 3A); this was apparent even with plants grown under control conditions (e.g., with no added stress or treatment; with reduced biomass as also noted before [18]). Accordingly, a more systematic study of lateral root development was conducted. While there were no significant differences in the lengths of the primary roots of *oxt6* mutant plants and those of wild-type plants (Fig. 2B), the number of lateral roots in the *oxt6* mutant was significantly reduced (p-value <0.05, Student's t-test, n≥12; Fig. 3A and 3B).

Lateral root development progresses through a characteristic series of stages, each defined by a series of cell divisions and expansions that lead to an emerged lateral root primordium. Upon close examination of the wild-type and mutant, lateral root primordia (LRP) could be seen in the *oxt6* mutant, and the cellular patterning and the number of cells of each stage were similar to the LRP in wild-type plants (Figure S2 in S1 File). However, the numbers of non-emerged LRPs in *oxt6* were lower than that in wild type (Fig. 3C). Closer examination revealed that fewer early-stage (stages I, II, and III) LRPs were found in the mutant compared with that of the wild-type (Fig. 3D). Taken together, these results are suggestive of a defect in early lateral root development in *oxt6*, such that fewer lateral root primordia are initiated; once initiation occurs, further development seems to proceed normally.

To confirm that the lateral root deficit seen in the *oxt6* mutant was due to a lack of AtCPSF30 expression, lateral root numbers on the C30G and C30GM plants was determined (Fig. 3B). As shown, the C30G plants had as many lateral roots as the wild-type; this indicates that the deficit seen in the *oxt6* mutant is dues to the ablation of the *OXT6* gene, and that the smaller of the two *OXT6* –encoded proteins is sufficient to restore wild-type lateral root development. In contrast, the C30GM plants, in which calmodulin binding domain was disrupted and had the primary root length as that of wild-type (Fig. 2B), retained the lateral root deficiency phenotype seen in the *oxt6* mutant (Fig. 3B). This result implicates the calmodulin-AtCPSF30 interaction in the functioning of AtCPSF30 in lateral root development.

### Reduced Fertility of *oxt6* Can Be Restored by the Calmodulin-binding Mutant Form of AtCPSF30

**Figure 4:**
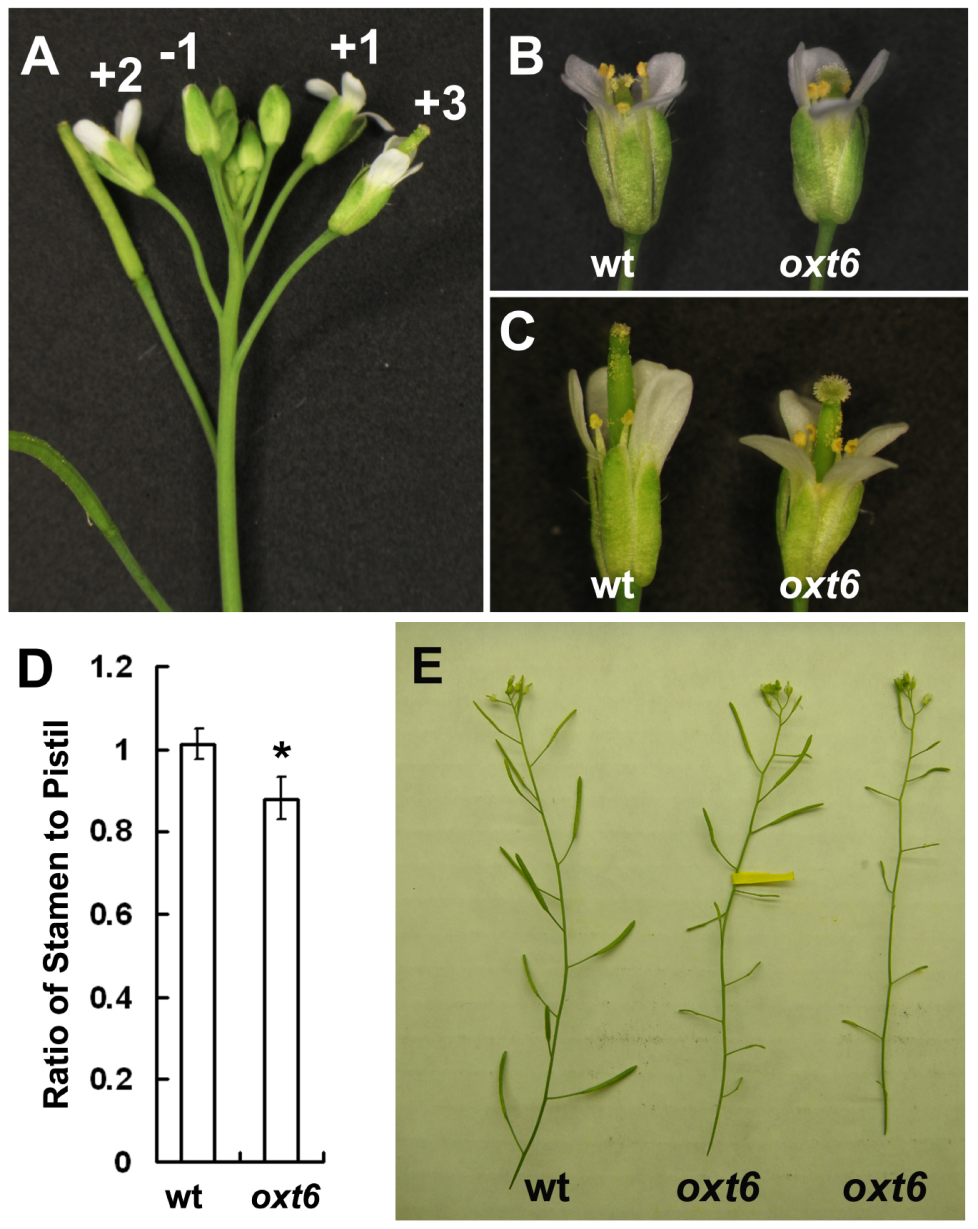
Characteristics of o*xt6* flowers. (A) A wild-type inflorescence showing selected stages of flower development. (B) and (C) flowers from wild-type and oxt6 plants at the +1 stage and +3 stage, respectively. (D) Lengths of the stamens at +1 stage, calculated as a fraction of the lengths of pistils (a value of 1.0 indicates that both organs are the same length). The difference between the wild-type and mutant was significant ate the p<0.05 level by the Student's t-test. (E) Silique phenotypes of the wild-type and *oxt6* mutant. A typical wild-type inflorescence is shown on the left, and an *oxt6* inflorescence on the right. The inflorescence in the middle is also from an *oxt6* plant; in this infloresence, the flowers indicated by the dark arrows were hand-pollinated with pollen from the same *oxt6* plant.

In the course of studying the *oxt6* mutant, it was noted that the mutant showed a diminished fertility, with flowers that form early (the first ten or so) having a reduced seed set. To explore the cause of the early fertility reduction of *oxt6* mutant, flowers from different developmental stages of wild type and *oxt6* were analyzed. In *Arabidopsis*, flowers develop continuously and can be identified in the order of developmental stages by their positions on the inflorescence [29]. Thus, flowers that have just opened are defined as +1 stage [30], the first unopened bud termed -1, the first opened flower +2, and so on, as shown in Fig. 4A. Up until the +1 stage (in floral organs up to and including the first unopened bud), there were no obviously morphological differences in floral organs between wild type and *oxt6*. At +1 stage in the wild-type (“wt” in Fig. 4B), pollen-bearing anthers were poised above the stigma, allowing pollen to fall on and fertilize the female gametophytes. At the corresponding stage of flower development in the *oxt6* mutant, anthers were not situated above the stigma (“*oxt6*” in Fig. 4B), a situation that is refractory to successful pollination. At the +3 stage, where the pollinated stigma had outgrown the anthers in wild-type, the anthers of the *oxt6* mutant remained below the stigma (Fig. 4C). A quantitative summary of this phenotype is provided in Fig. 4D.

To rule out the possibility that the reduced fertility of the *oxt6* mutant may be due to reduced pollen viability or function, two tests were conducted. In one, *oxt6* pollen was stained to test for viability; the results show that virtually 100% of the pollen grains were viable (Figure S3 in S1 File). In addition, *oxt6* pollen was used to pollinate *oxt6* stigmas from early flowers. Early flowers on *oxt6* plants that were pollinated with *oxt6* pollen formed elongated siliques (above yellow tag in Fig. 4E) that were comparable to those seen with wild-type flowers (“wt” in Fig. 4E). The seeds from these flowers were homozygous for the *oxt6* mutation. Thus, *oxt6* pollens are viable and functional in fertilization. These results suggest that the short stamen is responsible for the partial sterile phenotype of the *oxt6* mutant.

In contrast to the phenotype seen with the *oxt6* mutant, flowers that formed early on the *oxt6*::C30G plants were able to self-pollinate and subsequently develop normal siliques and seeds (Figure S4 in S1 File). Therefore, the altered floral organ growth and reduced fertility seen in the *oxt6* mutant are consequences of ablation of the AtCPSF30 gene, and that the wild-type AtCPSF30 protein is able to restore normal fertility. Interestingly, *oxt6*::C30GM plants also had wild-type floral organs and fertility (Figure S4 in S1 File). This result indicates that the interaction with calmodulin is not needed for the functioning of AtCPSF30 in flower development. Taken together, the experiments summarized in this subsection reveal that AtCPSF30 plays a subtle but important role in floral organ development, and they show that this function is not dependent on the interaction of AtCPSF30 with calmodulin.

### The *oxt6* Mutant Has Altered Responses to Several Plant Growth Regulators

**Figure 5:**
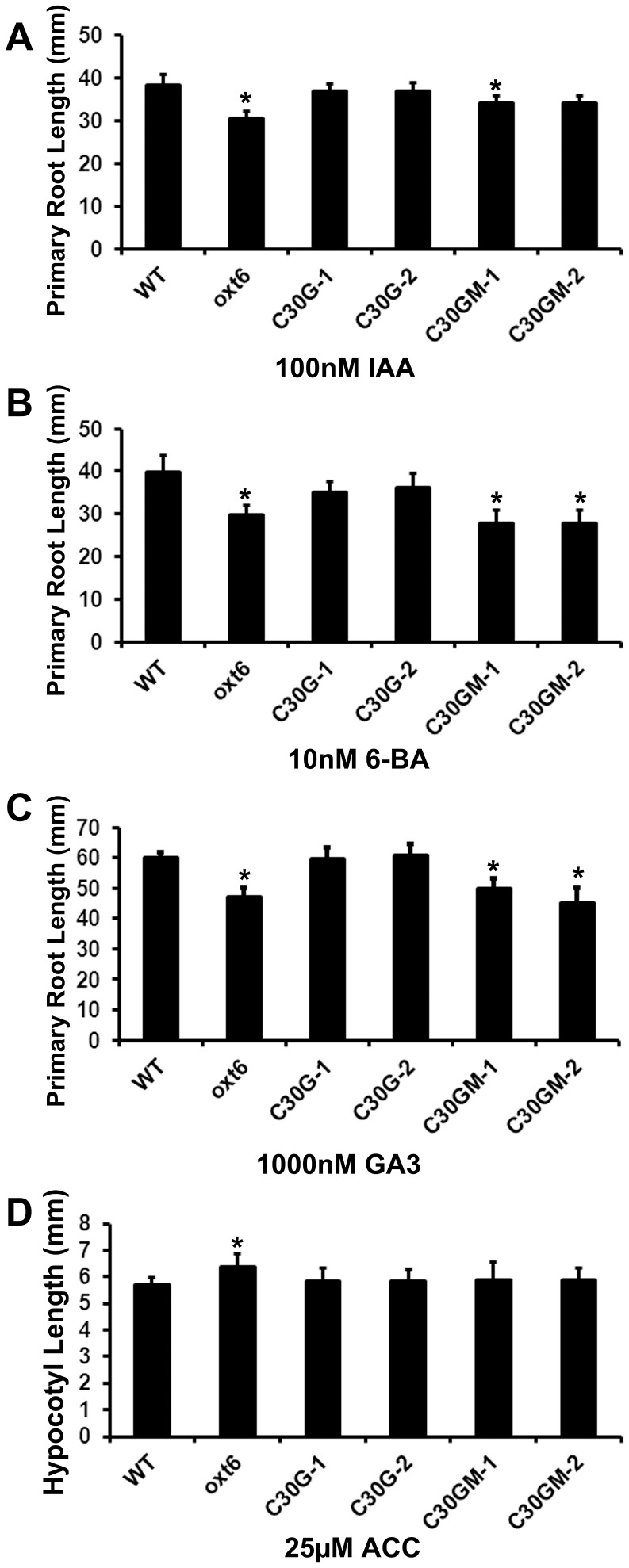
Responses of the wild-type, *oxt6* mutant, and complemented transgenic lines to a battery of hormones. The primary root length of wild-type, *oxt6*, and complemented lines was determined after 10 days of growth on half MS medium with the indicated concentrations of IAA (A), 6-BA (B), GA3 (C); the hypocotyl length on ACC (D). * indicates a statistically significant difference (p<0.05, Student's t-test) from the wild-type.

Previous research has shown that stamen filament elongation is associated with the functions of plant hormones such as auxin [31], [32], gibberellin [33], [34], and ethylene [35]. Lateral root formation is also a developmental process involving hormonal and environmental factors; for example, auxin transport system and signaling are essential to LR initiation and cytokinin negatively regulates LR formation [36], [37], [38]. It thus seemed possible that the *oxt6* mutant might have altered responses to plant growth regulators. This was tested using a standard root-length assay that is widely-used to measure the responses of *Arabidopsis* to hormones and other substances [39]. As shown in Fig. 5, the root length of *oxt6* mutant was significantly reduced when grown on media containing indoleacetic acid (IAA, an auxin; Fig. 5A), 6-benzylaminopurine (6-BA, a synthetic cytokinin; Fig. 5B), and gibberellic acid (GA3; Fig. 5C). Interestingly, the *oxt6* mutant was somewhat less sensitive to ACC (a precursor of ethylene) than the wild-type (Fig. 5D). The “no-hormone” controls for these experiments are shown in Fig. 2B and Figure S5 in S1 File.

The altered sensitivity of the *oxt6* mutant to IAA, GA3, 6-BA and ACC could all be reversed by C30G transgene (Fig. 5A–5D). This indicates that the phenotypes of the *oxt6* mutant are all due to the absence of AtCPSF30, and also that AtCPSF30 (as opposed to the larger isoform encoded by the *OXT6* gene) is sufficient for the wild-type responses to these four growth regulators. In contrast, the responses of *oxt6*::C30GM plants to IAA, GA3 and 6-BA were indistinguishable from the *oxt6* mutant (Fig. 5A–5C), thus showing that the CPSF30-calmodulin interaction is required for the CPSF30-mediated responses of plants to these substances. On the other hand, *oxt6*::C30GM plants were indistinguishable from the wild-type when treated with ACC (Fig. 5D), suggesting that the calmodulin binding domain is not involved in AtCPSF30-mediated responses to ethylene.

### The C30G and C30GM proteins restore wild-type poly(A) site choice to the *oxt6* mutant

**Figure 6:**
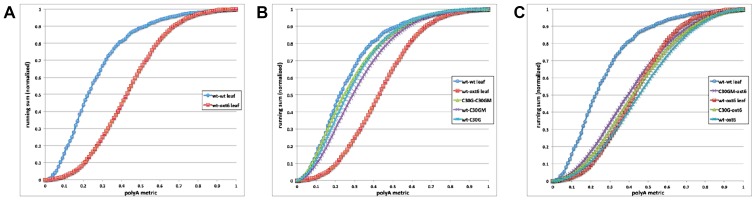
Global poly(A) site analysis of the four genotypes studied in this report. Poly(A) site distributions in extended 3′-UTRs were determined on a gene-by-gene basis and used for all possible pair-wise comparisons (wt-*oxt6*, wt-C30G, etc.) as described in Methods. Cumulative plots of the difference metric for each pairwise comparison were generated as described [25] and are shown here. A. Plot of data from [25], showing the results of comparisons for replicates from the same line (in this case, the wt; “wt-wt leaf”) the wt and *oxt6* mutant (“wt-oxt6 leaf”). These curves represent the expected “extremes” of similarity and differences, respectively. B. The comparisons involving the wt, C30G, and G30GM lines were superimposed on those shown in panel A. C. The three comparisons of the *oxt6* mutant with the other lines were superimposed on those shown in panel A.

The ability of the C30G and C30GM transgenes to restore wild-type responses of plants to MV (Fig. 2A) and ACC (Fig. 5D), and to restore wild-type fertility (Figure S4 in S1 File), indicates that the proteins encoded by these transgenes possess wild-type functionality insofar as they act in these different processes. However, it is possible that these roles are not related to that which AtCPSF30 (or, more properly, the two *OXT6*-encoded proteins) plays in mRNA 3′ end formation; for example, mRNA polyadenylation may require the presence of both *OXT6*-encoded proteins, and that AtCPSF30 may have additional roles apart from this process. To test this possibility, genome-wide poly(A) site choice in the *oxt6*::C30G and *oxt6*::C30GM lines was studied. For this, the approach described in Thomas et al. [25] was used; this involves the production and sequencing of short cDNA tags that query the mRNA-poly(A) junction, and subsequent computational analysis of poly(A) sites defined by these tags. Similar to Thomas et al., the relative usage of poly(A) sites in individual genes was estimated by enumerating the numbers of individual tags that define the respective sites, and differences in usage in the different strains (wt, *oxt6* mutant, and the *oxt6*::C30G and *oxt6*::C30GM lines) determined on a gene-by-gene basis. The output was a value (for each gene) that represents the difference between the two samples being compared; low numerical values denote genes that display little variability in poly(A) site choice, while high values (approaching 1.0, the upper limit allowed by the calculation) are indicative of substantial differences. The results for individual genes were then used to produce a genome-wide profile in which the fraction of all genes that possess comparison metrics below a given value was represented with a curve plotting the cumulative fraction of such genes against the comparison metrics. A typical set of results that shows the outcomes of comparisons of replicates from the same biological treatment (in this case, two wt samples) and of comparisons of the wt and *oxt6* mutant are shown in Fig. 6A (the values for these plots are those reported by Thomas et al. [25]); more similar samples will yield curves shifted to the left (as is the case for the wt-wt comparison) while different samples yield curves shifted to the right.

The results of comparisons of the wt with the *oxt6*::C30G and *oxt6*::C30GM lines are shown in Fig. 6B. As is apparent, all of the pairwise comparisons were much more coincident with the wt-wt comparison shown in Fig. 6A than with the wt-*oxt6* comparison. The converse was true for comparisons of the *oxt6* mutant with the wt and the *oxt6*::C30G and *oxt6*::C30GM lines (Fig. 6C); in these cases, there was much closer agreement with the wt-*oxt6* comparison than the wt-wt curve. The close agreement with the wt-wt curve seen in Fig. 6B and with the wt-*oxt6* curve in Fig. 6C indicates that, on a genome-wide basis, wild-type poly(A) site choice is altered in the roots of the *oxt6* mutant and is restored in *oxt6* transgenic lines that express the C30G or C30GM transgenes. The differences between the wt and *oxt6* mutant corroborate the results reported previously [25]. Moreover, the restoration of wt poly(A) site choice by the two transgenes indicates that both proteins (C30G and C30GM) are functional in mRNA 3′ end formation. This supports the proposition that many *oxt6*-related phenotypes that are restored by the C30G transgene are due to alterations of poly(A) site choice in the mutant.

Further analysis of poly(A) site choice in the different lines was conducted with the goal of identifying genes whose poly(A) site choice might be affected by the calmodulin-binding mutation in the C30GM transgene. For this the same poly(A) tag dataset was used, and screened for genes whose usage was wt-like in the C30G plants and *oxt6*-like in the C30GM plants. This computational exercise yielded no strong candidates (not shown). Thus, at the relatively gross level of whole roots, there seem to be few (if any) genes whose poly(A) site choice is affected by the calmodulin-binding site mutation.

### Genome-wide expression analysis in the wt and *oxt6* mutant

**Figure 7:**
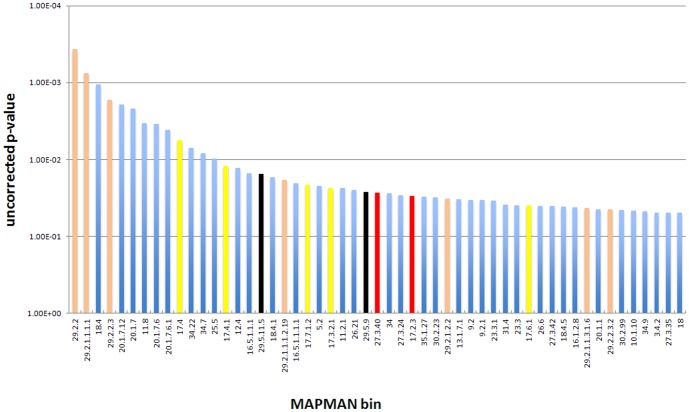
Results of an analysis of global gene expression in the roots of the wt and *oxt6* mutant. Gene expression was measured using the RNA-Seq functionality of CLC Genomics Workbench, and the results analyzed in MAPMAN as described in Methods. Bins representing functional groups of genes whose expression is significantly different in the wt and mutant were identified and the p-values that describe the conformance with the hypothesis that the two backgrounds are identical were plotted as shown; for this, the log(10) values for the reciprocal of each p-value was calculated and used in the graph. Bins mentioned in the text are color-coded and described beneath the plot. The full set of bins and p-values for this analysis is given in S2 Table.

The numerous phenotypes seen in the *oxt6* mutant suggest a widespread effect on gene expression. A prior microarray study conducted with RNA isolated from wt and *oxt6* leaves confirmed this suspicion [16]; in this study, numerous classes of genes whose expression was dependent on the presence of AtCPSF30 were identified. (These data were further used to establish a connection between AtCPSF30 and defense responses [17].) Among the classes of genes so identified (see Table S2 of ref. 16) were genes associated with defense responses, ubiquitin-mediated protein degradation, the encoding of cytoplasmic ribosomal proteins, and transporters. To further explore this, and to understand the nature of the root growth associated responses seen in this report (Figs. 2, 3, 5, and Figure S2 in S1 File), a comparable genome-wide study of gene expression in the roots of the wt and *oxt6* mutant was conducted. For this, the poly(A) tags used to assess poly(A) site choice were used to perform a gene expression analysis; this is possible because poly(A) tags are good proxies for mRNA abundance (much as are short cDNA tags used for RNA-Seq experiments [26]). Accordingly, expression analysis was done using the RNA-Seq tools in CLC Genomics Workbench. After removing genes whose expression was below 5 tpm in both samples, the results were used as input for the MAPMAN set of analysis tools [27], [28]. This set of tools allows for the identification of “bins” (groups of genes associated with various biological or molecular processes) that are affected in the *oxt6* mutant. The results (Fig. 7 and S1 Table) show that groups of genes associated with protein synthesis (primarily those that encode ribosomal proteins) and protein turnover are altered in the mutant; these results corroborate those described in Zhang et al. [16]. In addition, genes associated with hormone metabolism and with auxin responses are also affected by the *oxt6* mutation (Fig. 7). This is consistent with the many altered hormone responses and root phenotypes seen in the mutant. In addition, consistent with references 16 and 26, the expression of genes associated with defense responses was altered in the mutant (S1 Table).

This RNA-Seq analysis was expanded to include poly(A) tags isolated from the C30G and C30GM lines, so as to identify genes whose expression was affected specifically by the calmodulin binding site mutation in AtCPSF30. For this, the set of genes that passed the expression filter described in the preceding paragraph (11,564) were further filtered so that: the absolute values of the expression ratios in wt/*oxt6*, C30G/*oxt6*, wt/C30GM, and C30G/C30GM comparisons were greater than 2; and the absolute values of the expression ratios in C30GM/*oxt6* and wt/C30G comparisons was less than 2. This exercise yielded a list of 19 genes (S2 Table), three of them show relation to salicylic acid mediated signaling pathway, through which CPSF30 function is linked with defense response [17]. The connection between these genes and the calmodulin-dependent phenotypes described here (lateral root development and responses to auxin, cytokinin, and gibberellic acid) is not clear. Intriguingly, two genes were found: UBP16, Ubiquitin-specific protease 16, has a redundant role with UBP15 in cell proliferation, and plant development, such as root development, flower development [40]; MERI5B has xyloglucosyl transferase activity, which is critical to the cell wall formation; MERI5B is also associated with hormonal signaling, showing response to gibberellin stimulus [41]. None of these genes showed appreciable poly(A) site changes in the various lines (not shown), suggesting that the gene expression changes were likely secondary ones, not due to alterations in the processing of the respective pre-mRNAs.

## Discussion

### The Arabidopsis CPSF30 as an Integrator of Different Cellular Signaling Cues

Posttranscriptional controls are important determinants of regulated gene expression in plants. Among the steps that impact gene expression and plant phenotype is the process of 3′ end formation. This is exemplified by the isolation of mutants that have altered polyadenylation factor subunits; these mutants are affected in a broad range of physiological and molecular phenotypes, ranging from the timing of flowering to the termination of transcription [10], [11], [13], [14], [20], [42]. The results presented in the current study indicate that AtCPSF30 is one of the growing lists of plant polyadenylation factor subunits with roles in diverse molecular and physiological processes. Interestingly, the “reach” of AtCPSF30 is extensive, affecting aspects of floral organ and root development as well as responses to a range of growth regulators. However, extensive though this “reach” may be, it is also subtle. This is exemplified by the relatively restricted zone in the reproductive bolt that is affected by the *oxt6* mutation, as well as by the observations that, in spite of the altered responses of the *oxt6* mutant to several important growth regulators, the mutant is not severely impaired in growth and development.

Recalling that calmodulin inhibits RNA binding by AtCPSF30 in a calcium-dependent fashion [18], the results presented in this study suggest that regulated RNA binding by AtCPSF30 plays important roles in lateral root development, and in the responses of the plant to auxin, cytokinin, and gibberellic acid, and that this regulation is one mechanism by which calmodulin impacts these processes. While calcium and/or calmodulin have been linked, in various manners, with some of these processes or responses in plants [43], [44], [45], [46], it is not clear how the CPSF30-calmodulin interaction may contribute to the various phenotypes. Given that AtCPSF30 is a subunit of a core polyadenylation factor (CPSF), it stands to reason that the regulation of polyadenylation is a key component of these various developmental and physiological responses. Thus, it would seem as if calmodulin-regulated polyadenylation is an important contributing factor to a wide range of growth processes.

Interestingly, not all of the functions of AtCPSF30 in the cell require the interaction with calmodulin. This is true for the roles CPSF30 plays in floral organ growth as well as responses to oxidative stress and ethylene. This suggests that the activity of CPSF30 in the cell may be subject to regulation beyond that imparted by its interaction with calmodulin. While the nature of this regulation remains to be determined, one possible input may involve redox control. This follows from the fact that two cysteine residues in one of the three CCCH motifs of AtCPSF30 are engaged in a disulfide linkage [47], and that reduction of the disulfide bond inhibits the endonuclease activity of the protein [48]. Aspects of redox control have been implicated in all three of these aspects of plant development and growth [49], [50], [51], raising the possibility that redox regulation may involve AtCPSF30 in several developmental and physiological processes.

Just recently, the *oxt6* mutant was identified involving in salicylic acid-dependent signaling pathway leading to programmed cell death [17]. It was suggested that AtCPSF30 is required for disease resistance and immunity by mediating cell death through regulation of mRNA processing. This independent confirmation of the role of AtCPSF30 in signal transduction pathways in plants support our finding that it may integrate different signals to post-transcriptional gene expression regulations.

Based on these considerations, we propose a model whereby AtCPSF30 acts to integrate and transduce different signaling cues into different molecular and phenotypic outcomes. In this model, two different signaling inputs – Ca^2+^/calmodulin and redox-related disulfide bond rearrangements – act to inhibit different activities of AtCPSF30. Consequently, some aspects of mRNA 3′ end formation are altered, leading to changes in mRNA levels and/or functionality and subsequently in the levels and activities of strategic proteins. The aspect of mRNA 3′ end formation that is changed by inhibition of AtCPSF30 is likely to be the choice of poly(A) site in a transcription unit; this follows from the role of AtCPSF30 as a polyadenylation factor subunit [9], [18], [52].

### AtCPSF30, the Plant Polyadenylation Complex, and the Potential for Regulated Alternative Polyadenylation

AtCPSF30 is among the most enigmatic of the subunits of the plant polyadenylation complex. It binds RNA [18], [48], [52] and engages in protein-protein interactions with numerous other polyadenylation factor subunits [9], [52], [53], [54], [55]; several of these interactions are also seen in the yeast and mammalian polyadenylation complexes [3], [56], [57]. However, in yeast, the counterpart of CPSF30, Yth1p, is required for cell viability [58], [59]; this is not the case in Arabidopsis [16]. The reasons for this difference are not clear. It may be that plants possess other proteins that may be able to replace CPSF30 in the polyadenylation complex. In mammals, a related CCCH zinc finger protein, Smic1, may function in polyadenylation much as does CPSF30 [60], [61]. The Arabidopsis genome possesses genes whose predicted products show some relatedness with Smic1 [62], thus lending credence to this possibility. Like AtCPSF30, the possible Arabidopsis Smic1 ortholog (AtSmic1) possesses RNA-binding and ribonuclease activities [62]; however, other biochemical or functional parallels between AtSmic1 and AtCPSF30 have not been explored.

Alternatively, the plant polyadenylation complex may differ in subtle but important ways insofar as CPSF30 functioning is concerned, such that the essential function(s) of Yth1p in yeast are accounted for by other subunits of the plant polyadenylation complex, or are altogether absent in the plant complex. Yth1p possesses an array of five CCCH zinc finger motifs, whereas AtCPSF30 (and all other plant CPSF30 orthologs) possess but three such motifs. The three plant motifs are related in sequence and function to the 2^nd^, 3^rd^, and 4^th^ (“reading” from the N- to C- terminus of the protein) of Yth1p. Thus, it is possible that functions associated with the 1^st^ and/or 5^th^ CCCH motifs of Yth1p may be essential in yeast, but that these functions have been co-opted by other subunits of the plant complex, or even lost in the course of evolution. In either case, this structural difference may underlie the different requirements for growth in yeast and plants.

Regardless of the underlying reasons, the non-essential nature of AtCPSF30 is probably important in its role as a regulatory conduit. As discussed in the preceding subsection, different possible signaling inputs inhibit AtCPSF30; the fact that AtCPSF30 is not essential provides an explanation for how such signaling inputs may inhibit ACPSF30 without dramatically impairing overall developmental and physiological responses. Thus, AtCPSF30 may promote or inhibit usage of a subset of poly(A) sites in the Arabidopsis genome, but make little contribution to other sites (perhaps the majority of poly(A) sites). For most genes, poly(A) site changes associated with inhibition of AtCPSF30 activity would have modest effects on mRNA functionality. Only in those instances where an altered poly(A) site changes the protein-coding capacity of the mRNA (by, for example, polyadenylation within an upstream intron) or the stability of the mRNA (for example, by introducing or removing microRNA target sequences) would a significant contribution to overall gene expression levels be manifest. Indeed, our recent work has indicated that there is a significant level of alternative polyadenylation in the *oxt6* mutant in contrast to that of the wild-type [25].

In summary, the studies presented in this report indicate that the Arabidopsis polyadenylation factor subunit AtCPSF30 has roles in a number of different developmental processes and responses to stimuli. Moreover, they show that the interaction between AtCPSF30 and calmodulin is required for some of these roles, but not for others. These results reveal AtCPSF30 to be a multifaceted integrator of multiple signaling inputs as well as a transducer that coordinates cellular signals with a number of important physiological outcomes.

## Supporting Information

S1 Table MAPMAN analysis of gene expression in the wt and *oxt6* mutant. One sheet has a list of all genes that pass the expression filter (5 tpm in at least one of the four samples), and the normalized and transformed expression values for these. The second sheet has the results of the Wilcoxon Rank Sum Test, listing the uncorrected p-values returned for all MAPMAN bins. This sheet also has the same plot shown in Fig. 7, showing the source of the values in this graph.
(XLSX)

S2 Table Genes whose expression are affected by the mutation in the calmoduin-binding site of AtCPSF30.
(XLSX)

S1 File Figure S1, qRT-PCR analysis of transgene expression in the different transgenic plant lines. Figure S2, Lateral root primodium (LRP) development in the WT and *oxt6* mutant. Figure S3, Alexander staining of *oxt6* pollen. Figure S4, Inflorescences from *oxt6*::C30G and *oxt6*::C30GM transgenic plants. Figure S5, Hypocotyl length of Wt, *oxt6*, C30G, and C30GM plants on MS plate.
(PDF)
