# Supplementary material for: Integration of Developmental and Environmental Signals via a Polyadenylation Factor in Arabidopsis
Source: PLoS One. 2014 Dec 29;9(12):e115779. doi: 10.1371/journal.pone.0115779 (PMC4278772; doi:10.1371/journal.pone.0115779)
Supplement: S1 File — Figure S1, qRT-PCR analysis of transgene expression in the different transgenic plant lines. Figure S2, Lateral root primodium (LRP) development in the WT and oxt6 mutant. Figure S3, Alexander staining of oxt6 pollen. Figure S4, Inflorescences from oxt6::C30G and oxt6::C30GM transgenic plants. Figure S5, Hypocotyl length of Wt, oxt6, C30G, and C30GM plants on MS plate. (PDF) [file pone.0115779.s003.pdf]

Liu et al. Supplementary Data

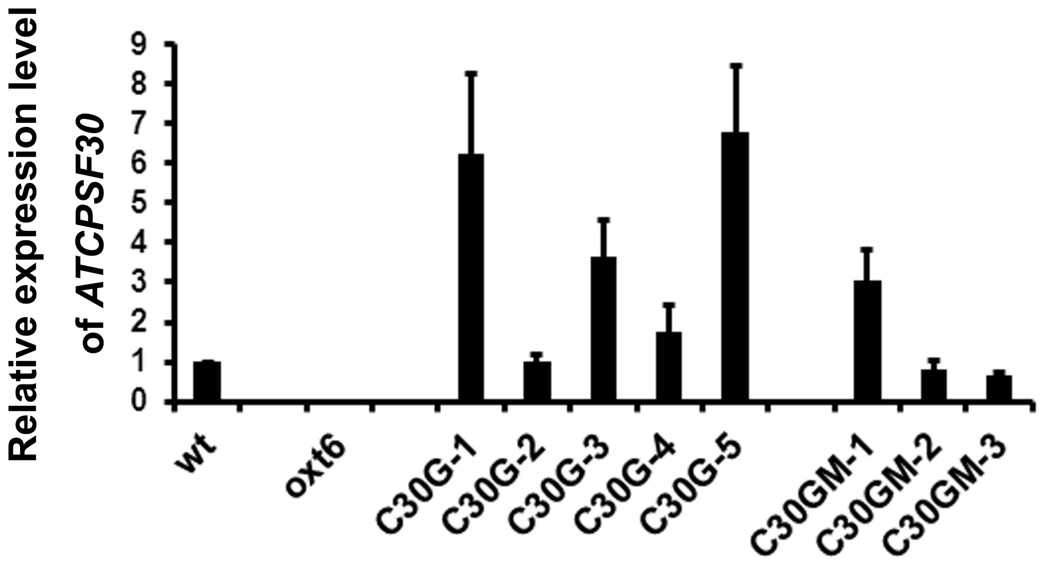

Figure S1. Quantitative RT/PCR determination of CPSF30 gene expression in the wild-type, *oxt6* mutant, and individual complemented transgenic plants lines. cDNA was prepared and PCR performed as described in the Methods; the *TIP41-LIKE* was used for an internal standard. Values were normalized such that expression in the wild-type was 1.0. Individual *oxt6*::C30G are noted with the designations C30G-1, C30G-2, etc., and *oxt6*::C30-GM lines with the designations C30GM-1, C30GM-2, etc.

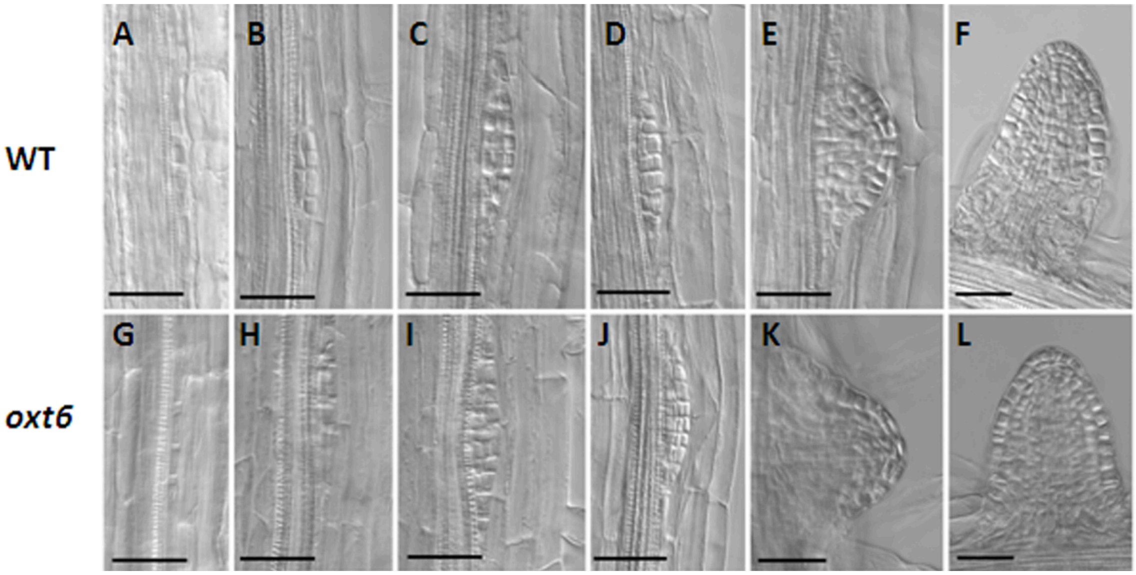

Figure S2. Lateral root primodium (LRP) development in WT and *oxt6* mutant. Nomarski images of LRP in primary roots in wild-type (top panel, A-F) and *oxt6* mutant (lower panel, G-L). A to D are at stages I, II, III and IV in wild-type. E and F show the LRPs that beyond stage IV and fully emerged LR, respectively. G to L represent the LRPs in *oxt6* mutant that have the similar stages as those in A to F, which are similar to the LRPs in wild-type in the cellular patterning and the number of cells. Bar=50μm.

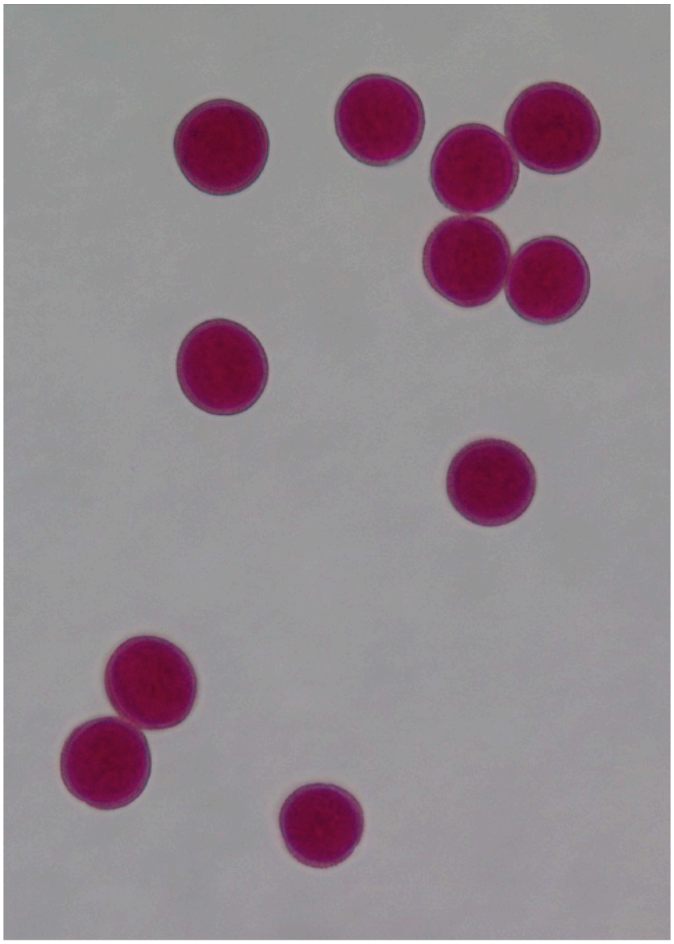

Figure S3. Alexander staining of *oxt6* pollen. Pollen were collected from an *oxt6* mutant and stained as described previously (Johnson-Brousseau and McCormick, 2004).

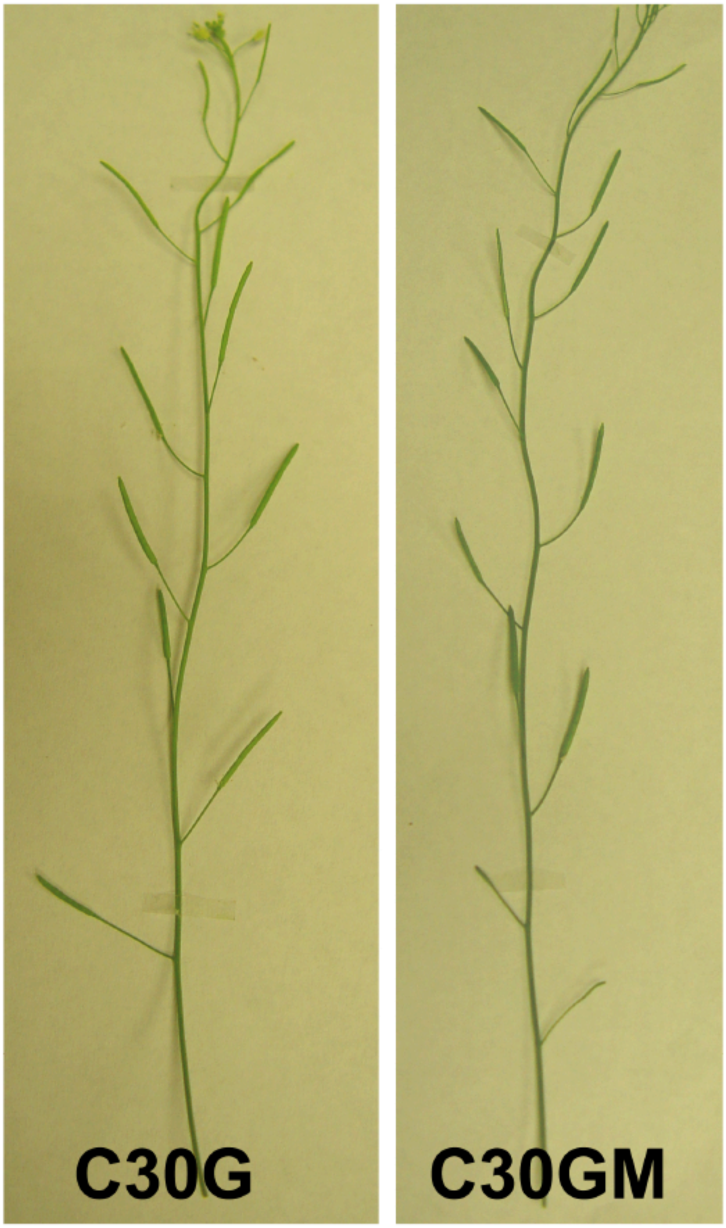

Figure S4. Inflorescences from *oxt6*::C30G (**A**) and *oxt6*::C30GM (**B**) transgenic plants, which were 6 weeks old, and grown in the soil with 16h light/8h dark.

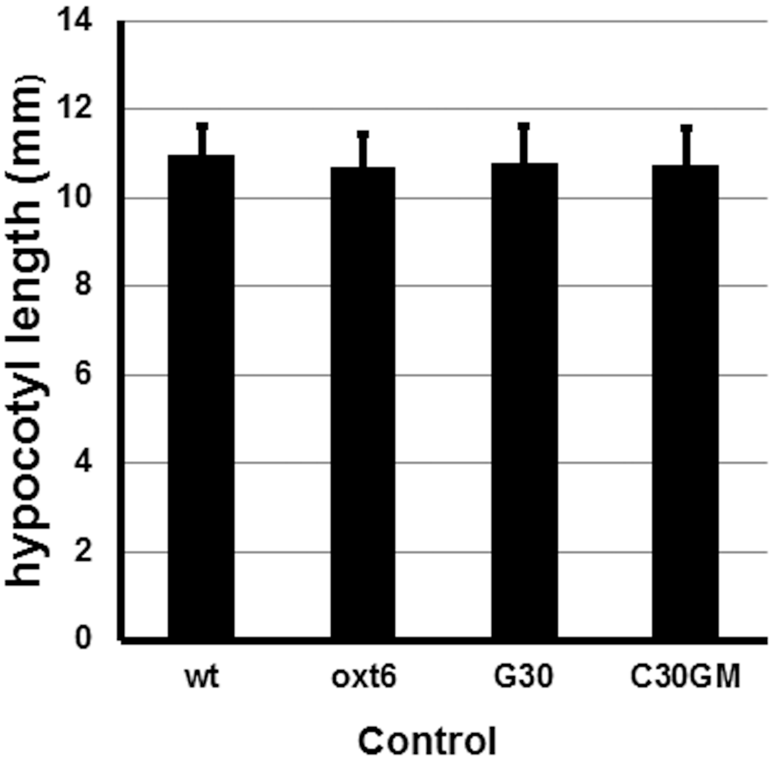

Figure S5. Hypocotyl length of Wt, *oxt6*, C30G, and C30GM plants on MS plate. The growth conditions and measurement are as in Figure 2, but without MV in the medium.
